# Acute Coronary Syndrome in Patients with Prior Coronary Artery Bypass Surgery: Observations from a 20-Year Registry in a Middle-Eastern Country

**DOI:** 10.1371/journal.pone.0040571

**Published:** 2012-07-18

**Authors:** Rafid Al-Aqeedi, Nidal Asaad, Awad Al-Qahtani, Rajvir Singh, Hajar A. Al Binali, Abdul Wahid Al Mulla, Jassim Al Suwaidi

**Affiliations:** Department of Cardiology and Cardiovascular Surgery, Heart Hospital Hamad Medical Corporation (HMC), Doha, Qatar; S.G. Battista Hospital, Italy

## Abstract

**Objectives:**

Clinical characteristics and trends in the outcome of acute coronary syndrome (ACS) in patients with prior coronary artery bypass graft surgery (CABG) are unclear. The aim of this study was to evaluate clinical characteristics, in-hospital treatment, and outcomes in patients presented with ACS with or without a history of prior CABG over 2 decades.

**Methods:**

Data were derived from hospital-based study for collected data from 1991 through 2010 of patients hospitalized with ACS in Doha, Qatar. Data were analyzed according to their history of prior CABG. Baseline clinical characteristics, in-hospital treatment, and outcome were compared.

**Results:**

A total 16,750 consecutive patients with ACS were studied, of which 693 (4.1%) had prior CABG. Patients with prior CABG were older (mean 60.5±11 vs. 53±12 years; P = 0.001), more likely to be females and have more cardiovascular risk factors than the non-CABG group. Prior CABG patients had larger infarct size, were less likely to receive reperfusion therapy, early invasive therapy and more likely to receive evidence-based therapies when compared to non-CABG patients. In-hospital mortality and stroke rates were comparable between the 2 groups. Over 2 decades, there was reduction in the in-hospital mortality rates and stroke rates in both groups (CABG, death; 13.2% to 4%, stroke; 1.9% to 0.0%, non-CABG, death; 10% to 3.2%, stroke 1.0% to 0.1%; all, p = 0.001).

**Conclusion:**

Significant reduction in-hospital morbidity and mortality among ACS patients with prior CABG over a 20-year period.

## Introduction

There is a growing global attention concerning the short and long term prognosis of acute coronary syndrome (ACS) in patients with prior coronary artery bypass grafting (CABG). Despite the indisputable benefit of CABG surgery in reducing morbidity and mortality [1], acute myocardial infarction (MI) still reported with an incidence of 3% to 8% annually following bypass graft surgery [2]. In patients with ACS, some of the previous reports suggested prior CABG as an independent risk factor for mortality [3], [4], while others reported equal or even more favorable prognosis when compared to non-BABG patients [5]–[9]. A recent global rising in the number of ACS was observed in patients with prior CABG [10], as a result of the worldwide increasing numbers of bypass surgeries performed annually [11], [12] and in the consequences of the angiographically recognized loss of vein graft after surgery (50% become diseased and 25% occluded) by 5 years [2].

In general, patients with prior CABG have often been underrepresented in ACS clinical trials. Furthermore, trend in the outcome of ACS patients with prior CABG patients is lacking. We hypothesize that the clinical characteristics, treatment and outcome of ACS patients with prior CABG to be different than patients without prior CABG. Our second hypothesis is that the improvement in the surgical techniques of CABG and increase use of evidence-based therapy over the past two decades resulted in significant reduction in morbidity and mortality of ACS patients with prior CABG.

## Materials and Methods

### Study Setting

This study is based at Hamad General Hospital, Doha, Qatar. This hospital provides inpatient and outpatient medical and surgical care for all population of Qatar, including nationals and expatriates. More than 95% of cardiac patients are being treated in this hospital, making it an ideal center for population-based studies. The vast majority of acute coronary syndrome patients (>95%) are admitted at this hospital. In the last decade of the 20^th^ century, cardiovascular diseases are the leading causes of morbidity and mortality in Qatar. Qatar is a small country with a population of around 600,000 (2001 Census) and 1,6 million (2010 Census), consisting of Qatari and other Middle Eastern Arabs (less than 40%) and non-Middle Eastern Arabs of which the vast majority are South Asians mainly from India, Pakistan, Nepal and Bangladesh.

The Cardiology and Cardiovascular Surgery Database at Hamad General Hospital was used for this study. A case report form with a specific registration identification number for each patient admitted to Hamad General Hospital with cardiac illnesses was filled out by the assigned physician who followed the patient throughout hospital stay using standard definitions, and completed before the patient’s hospital discharge. Data were collected from the clinical records according to predefined criteria for each variable. These records have been coded and registered at the cardiology department since January 1991 [13]. With the described database, all patients presenting with ACS whom hospitalized in the 20-year period between January 1991 and end of 2010 were retrospectively identified. We categorized our study cohort (n = 16,750) on the basis of prior bypass surgery into two groups, with or without prior CABG. Accordingly data were analyzed and compared for clinical characteristics, treatment and in-hospital outcomes. Ethical approval from Research Committee of Hamad Medical Corporation was obtained before starting collection of data for the study. The ethics committee waived the need of informed consent because of its retrospective analysis and the fact that the data was analyzed anonymously.

### Definitions

Standard definitions were used to diagnose ACS. Formerly acute MI was defined for this study according to the World Heart Organization criteria for Q-wave and non Q-wave MI. Then, STEMI and NSTEMI were defined by a positive serial troponin-T blood test result (≥0.1 ng/ml) in the setting of symptoms and electrocardiographic changes consistent with MI [14]. Unstable angina was diagnosed if the patient had a negative cardiac biomarker and any one of the following characteristics: new-onset angina (<2 months) of at least class III according to the Canadian Cardiovascular Society, prolonged (>20 minutes) angina at rest, recent (<2 months) worsening of angina pectoris, or angina that occurred within two weeks of an acute MI [15].

The presence of hypertension was determined by any documentation in the medical record of hypertension or if the patient was on treatment by the patient’s physician. The presence of diabetes mellitus was determined by the documentation in the patient’s previous or current medical record of a documented diagnosis of diabetes mellitus that had been treated with medications or insulin. Smoking history: Patients were divided into current cigarette smokers, past smokers defined as more than 6 months abstinence from smoking, and those who never smoked. Chronic renal impairment was defined as creatinine >1.5 upper normal range. The presence of hyperlipidemia was determined by the demonstration of a fasting cholesterol >5.2 mmol/L in the patient’s medical record, or any history of treatment of hyperlipdemia by the patient’s physician. Congestive heart failure (CHF) was defined using the Framingham criteria. The simultaneous presence of at least two major criteria or one major criterion in conjunction with two minor criteria was required to establish a diagnosis of CHF. Major criteria included paroxysmal nocturnal dyspnea or orthopnea, jugular venous distension, pulmonary rales, radiographic cardiomegaly, acute pulmonary edema, a third heart sound, central venous pressure above 16 cm of water, hepatojugular reflux, and weight loss of at least 4.5 kg in 5 days in response to treatment of heart failure. Minor criteria included bilateral ankle edema, nocturnal cough, dyspnea on ordinary exertion, hepatomegaly, pleural effusion, and a heart rate of at least 120 beats per minute. Minor criteria were acceptable only if they could not be attributed to any other medical condition (such as chronic lung disease, cirrhosis, ascites, or the nephrotic syndrome) [16].

### Statistical Analysis

Patients’ characteristics are presented in frequency and percentages for categorical variables and in mean ± SD for continuous variables. The frequencies of categorical variables in the two populations (CABG and no CABG) were compared using the Chi-square test and continuous variables were compared using the two-tailed Student’s *t* test or Mann Whitney U test wherever applicable. Variables influencing in-hospital mortality were assessed with multiple logistic regressions enter method. Odds ratios (RR), 95% CI, and *p* values were reported for significant predictors. A *p* value of less than 0.05 was considered statistical significant. All *p* values were the results of two-tailed tests. Data analyses were carried out using the Statistical Package for Social Sciences version 18.0 (SPSS Inc., USA).

## Results

### Study Population

Overall, from January, 1991 to the end of year 2010, a total of 41,438 patients hospitalized with acute cardiac disease were scrutinized 16,750 of these patients were admitted with ACS, of them 693 patients (4.1%) were with prior CABG and 16,057 patients without previous CABG.

### Baseline Clinical Characteristics

Table 1 shows the baseline clinical characteristics of the patients. Those patient with prior CABG were older than patients without prior CABG (mean 60.5±11 vs 53±12 years; P = 0.001). Females constituted 17.7% of the CABG group and were significantly more in numbers than the non-CABG group 14.7% (P = 0.03). The body mass index mean values showed no significant variation between the two groups (27.5±5 vs 28±14; P = 0.61). Study of different population ethnicity showed Middle Eastern Arabs were significantly more common to have a prior bypass surgery than patient without bypass (55.4% vs 41.9%; P = 0.001). While South Asian population were less commonly had prior CABG (35.4% vs 45.1%; P = 0.001). Sub-analysis of age revealed patients with prior CABG were significantly older than their counterparts in all ethnicities (p = 0.001).

**Table 1:** Acute coronary syndrome patients’ baseline demographics, clinical characteristics and outcomes according to their history of prior coronary artery bypass surgery.

Variable	Prior CABG (n = 693)	No CABG (n = 16057)	P Value
**Patient characteristics at admission** (%)			
Age in year (mean ± SD))	60.5±11	53.7±12	0.001
Female gender	17.7	14.7	0.03
Body mass index (kg/m^2^) (mean ± SD)	27.5±5	28±14	0.61
**Ethnicity** (%)			
Middle Eastern Arabs	55.4	41.9	0.001
South Asians	35.4	45.1	0.001
Others	9.2	13.0	0.001
**Age in different ethnicities** (mean ± SD)			
Middle Eastern Arabs age	63.7±10	58.8±13	0.001
South Asians	54.5±8	49.2±9	0.001
Others	63.5±12	53.0±11.5	0.001
**Cardiovascular risk factors** (%)			
Current smoker	20.9	33.9	0.001
Hypertension*	60.6	40.4	0.001
Diabetes mellitus†	59.9	40.8	0.001
Chronic renal impairment	8.1	3.1	0.001
Dyslipidemia††	26.7	21.0	0.001
**Prior cardiovascular disease** (%)			
Prior myocardial infarction	43.3	15.5	0.001
Prior heart failure	14.6	7.6	0.001
Prior or current atrial fibrillation	2.2	1.7	0.37
**In-hospital procedure/therapy** (%)			
Rate of thrombolysis‡	8.8	29.2	0.001
Coronary angiography	18.0	20.9	0.07
Percutaneous coronary intervention	5.1	10.6	0.001
Peak CK-MB (mean ± SD)	87.4±288	231.2±757	0.001
**Left ventricular ejection fraction** (%)			
Normal: LVEF of ≥55%	15.2	20.1	0.02
Mild: LVEF of 40%–54%	41.4	47.1	
Moderate: LVEF of 30%–39%	27.7	20.7	
Severe: LVEF of <30%	17.8	12.2	
**Hospital days** (mean ± SD)			
CCU stay	4.6±5.6	3.5±3.2	0.006
Total hospital stay	6.7±7	5.5±5.5	0.001
**In hospital outcome** (%)			
Death	5.8	5.2	0.52
Stroke	0.4	0.3	0.53

Data are expressed in numbers (%) of patients unless otherwise indicated.

* Systolic blood pressure >140 mm Hg, diastolic blood pressure >90 mm Hg, or current antihypertensive treatment.

† Patient had been informed of the diagnosis by a physician before admission and for type 1 or 2 diabetes.

†† Total cholesterol >5.2 mmol/L or current use of lipid-lowering agent.

‡ Of patients eligible for thrombolysis (ST-elevation myocardial infarction (previously known Q wave MI) or new or presumed left bundle branch block).CABG  = coronary artery bypass graft; CCU = coronary care unit; MI = myocardial infarction; STEMI = ST elevation myocardial infarction; NSTEMI = non ST elevation myocardial infarction.

When compared to patients without CABG, those with prior CABG had more adverse baseline characteristics. They were more likely to have hypertension (60.6% vs 40.4%; P = 0.001), diabetes mellitus (59.9% vs 40.8%; P = 0.001) and dyslipidemia (26.7% vs 21%; P = 0.001). In addition, they tended to have more prior congestive heart failure (1.64% vs 7.6%; P = 0.001) and chronic renal impairment (8.1% vs 3.1%; P = 0.001). Whereas patients with prior bypass surgery were less likely to be a current smokers (20.9% vs 33.9%; P = 0.001). Furthermore, patients with prior bypass showed a greater preponderance of old MI (43.3% vs 15.5%; P = 0.001).

### In-hospital Management

In STEMI patients, thrombolytic therapy was used as a primary reperfusion therapy. There was a significant higher rate of thrombolysis in patients without prior CABG (29.2% vs 8.8%; P = 0.001). Likewise, coronary angiography and percutaneous coronary intervention (PCI) as a therapeutic mode for ACS, was showed a significant more frequency in patients without prior CABG than patients with prior CABG (10.6% vs 5.1%; P = 0.001). In patients with prior CABG, the pre-discharge left ventricular ejection fraction (LVEF) was more frequent ≤30% (severe LV dysfunction); (17.8% vs 12.2%; P = 0.02) and more common between 30–39% (moderate LV dysfunction); (27.7% vs 20.7%; P = 0.02) as compared to patients without prior CABG. In contrary to that normal LVEF (≥55%) and mild LV dysfunction (LVEF = 40–54%) were more prevalent in patient without prior bypass graft surgery (20.1% vs 15.2%, 47.1 vs 41.4; P = 0.02, respectively).

During hospitalization, the comparison of coronary care unit days of stay showed a significant longer stay in CABG patients as compared to patients without prior CABG (4.62±5.6 vs 3.46±3.2; p = 0.006). Likewise, total days of stay including step-down ward stay was longer in CABG patients (6.69±7 vs 5.49±5.54; p = 0.001). Patients with no prior bypass graft surgery reported higher mean values of peak ceatine kinase-MB when presented with ACS (231.2±757 vs 87.4±288; p = 0.001). Similarly, higher positive results of troponin-T during hospitalization (43.3 vs 36.2; p = 0.001).

### Medications Prescribed

Table 2 shows medication use before, during admission and at discharge in ACS patients without prior CABG.

**Table 2:** Medication received before, during admission and at discharge in acute coronary syndrome patients with or without prior coronary artery bypass surgery.

Medications	Before Admission			At Admission			At Discharge		
	Prior CABG (n = 693)	No CABG (n = 1605)	P value	Prior CABG (n = 693)	No CABG (n = 16057)	P value	Prior CABG (n = 693)	No CABG (n = 16057)	P value
**Aspirin**	75.0	30.6	0.001	90.8	90.6	0.869	86.0	89.2	0.008
**Clopidogrel**	21.1	9.9	0.001	32.8	33.3	0.787	33.9	34.4	0.782
**B blocker**	27.3	13.1	0.001	41.7	49.7	0.001	28.6	30.7	0.23
**CCB**	10.2	4.3	0.001	17.2	8.0	0.001	22.5	11.5	0.001
**ACE inhibitors/ARBs**	20.8	10.7	0.001	36.4	30.6	0.001	42.3	40.1	0.24
**HMG-CoA reductase inhibitor**	–	–	–	–	–	–	59.0	51.5	0.001
**GPIIb/IIIa inhibitors**				4.2	4.5	0.726			
**Unfractionated heparin**				31.3	38.6	0.001			
**LMWH (enoxaparin)**				21.1	18.8	0.141			

Data are expressed in numbers (%) of patients.CABG  =  coronary artery bypass graft; HMG-CoA  =  hydroxy methyl glutaryl-coenzyme A; GP  =  glycoprotein; LMWH  =  low molecular weight heparin; CCB  =  calcium channel blockers; ACE = angiotension converting enzyme inhibitor, ARB = angiotensin receptor blocker.

#### Before admission

Primarily ACS patients with prior CABG were more commonly prescribed previous treatment with aspirin, clopidogrel, beta blockers, calcium channel blockers (CCB) and angiotensin converting enzyme (ACE) inhibitors/angiotensin receptor blockers (ARB) than patients with no prior CABG (P = 0.001).

#### During hospital admission

There were no significant differences between the two groups in the medications provided, aspirin (90.8 vs 90.6; p = 0.869), clopidogrel (32.8 vs 33.3; p = 0.787), Glycoprotien IIb/IIIa inhibitors (32.8 vs 33.3; p = 0.787) and low molecular weight heparin (enoxaparin), (21.1 vs 18.8; p = 0.141), but patients with prior CABG were less likely prescribed beta-blockers (41.7% vs 49.7%; P = 0.001) and unfractionated heparin (31.3 vs 38.6; p = 0.001). ACE inhibitors/ARBs and CCBs were prescribed more in patients with prior CABG (36.4% vs 30.6%; P = 0.001, 17.2 vs 8.0; p = 0.001) respectively.

#### At discharge

There was no significant difference in clopidogrel (33.9 vs 34.4; p = 0.782), β-blockers (28.6 vs 30.7; p = 0.24) and ACE inhibitors/ARBs prescriptions (42.3 vs 40.1; p = 0.24), but patients with prior CABG were furthermore less prescribed aspirin (86.0% vs 89.2%; P = 0.008) while, CCB and HMG-CoA reductase inhibitors were more likely prescribed in these patients (22.5% vs 11.5%; P = 0.001 and 59.0% vs 51.5%; P = 0.001, respectively).

### Outcomes

The in-hospital mortality rates observed no significant difference in patients with prior CABG compared to those without prior CABG (5.8% vs 5.2%; P = 0.52), and stroke as a complication was also not different (0.4% vs 0.3%; P = 0.53).

#### Trend of outcomes

**Figure 1:**
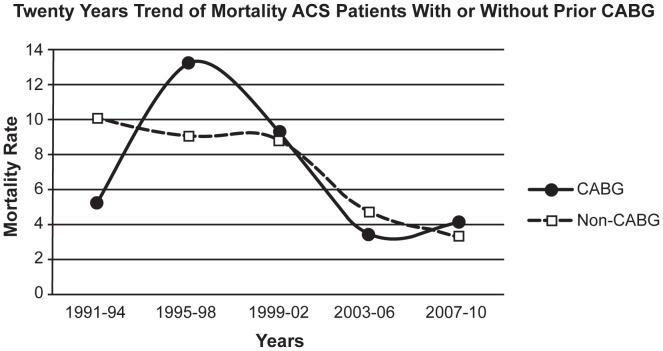
The 20 years trend of mortality in 16,750 patients with acute coronary syndrome with or without history of prior coronary artery bypass surgery. CABG  =  coronary artery bypass graft; ACS  =  acute coronary syndrome.

Table 3 shows the trend of outcomes in term of mortality and stroke in ACS patients with or without prior CABG. Over the 20-year period, there was significant increasing number of CABG operations (p = 0.001). The in-hospital mortality rate significantly reduced from peak 13.2% to 4.0% (p = 0.001) for patients with prior CABG and from 10% to 3.2% (p = 0.001) for patients without prior CABG (figure 1). Stroke rates also, reduced from peak 1.9% to 0.0% (p = 0.001) for patients with prior CABG and from peak 1.0% to 0.1% (p = 0.001) for patients without prior CABG.

**Table 3:** Trend of outcomes (1990 to 2010), include mortality and stroke in acute coronary syndrome patients with or without prior coronary artery bypass surgery.

Outcomes	1991–94	1995–98	1999–02	2003–06	2007–10	P value
**No. of CABG/ACS**	60/1775 3.4%	53/1836 2.9%	86/2412 3.6%	243/4652 5.2%	251/6061 4.1%	0.001
**Death in CABG**	3/60 5.0%	7/53 13.2%	8/86 9.3%	8/243 3.3%	10/251 4.0%	0.02
**Death in non CABG**	172/1715 10%	161/1783 9%	205/2526 8.8%	204/4412 4.6%	185/5821 3.2%	0.001
**No. of stroke/ACS**	16/1775 0.9%	18/1836 1.0%	12/2412 0.5%	5/4652 0.1%	6/6061 0.1%	0.001
**Stroke in CABG**	0/60 0%	1/53 1.9%	0/86 0%	1/243 0.4%	0/251 0%	0.001
**Stroke in non CABG**	16/1751 0.9%	17/1783 1.0%	12/2326 0.5%	4/4412 0.1%	6/5821 0.1%	0.001

Data are expressed in numbers (%) of patients. CABG  =  coronary artery bypass graft; ACS  =  acute coronary syndrome.

#### Trend of medications

Table 4 shows medications given to patients with ACS during admission. A significant increase in the use of evidence-based therapies on admission was observed during the span of 20 years including, aspirin (77.2% to 95.1%, p = 0.001), beta blockers (31% to 64%, p = 0.001), ACE inhibitors/ARBs (12.7% to 45.1%, p = 0.001) and clopidogrel (17.9% to 77.9%, p = 0.001).

**Table 4:** The 20-year trend of medications prescribed during admission in patient with acute coronary syndrome.

Medications	1991–94	1995–98	1999–02	2003–06	2007–10	P value
**Asprin**	1371/177577.2%	1508/183682.1%	2153/241289.3%	4367/465593.8%	5774/607295.1%	0.001
**B-blocker**	550/177531.0%	642/183635%	961/241239.8%	2235/465548%	3884/607264%	0.001
**ACE inhibitors/ARBs**	226/177512.7%	319/183617.4%	464/241219.2%	1421/465530.5%	2741/607245.1%	0.001
**Clopidogrel**	0/17750%	0/18360%	0/24120%	1421/465517.9%	4733/607277.9%	0.001

Data are expressed in numbers (%) of patients. Same abbreviations mentioned in table 2.

#### Ten years comparison of outcomes

Table 5 reveals morbidities and mortality comparison in periods, 1991-2000 and 2001-2010 according to history of prior CABG. There was significant reduction in mortality and the majority of morbidities measured (reinfarction, cardiogenic shock and advanced heart block) in the latter when compared to the earlier time periods regardless of prior CABG history, with the exception of stroke and ventricular arrhythmia rates which were comparable in the two time periods among patients with prior CABG only.

**Table 5:** Trend of morbidities and outcomes with comparisons for 1991–2000 and 2001–2010 periods, in patients with acute coronary syndrome.

Mortality/Morbidities	Prior CABG (n = 693)		P value	No CABG (n = 16057)		P value
	1991–2000 (n = 155)	2001–2010 (n = 538)		1991–2000 (n = 4660)	2001–2010 (n = 11397)	
**Deaths**	14(9)	22(4.1)	0.02	451(9.7)	476(4.2)	0.001
**Stroke**	1(0.6)	1(0.2)	0.35	43(0.9)	12(0.1)	0.001
**Cardiogenic shock**	13(8.4)	12(2.2)	0.001	187(4.0)	251(2.2)	0.001
**Re-infarction**	5(3.2)	0(0)	0.001	76(1.6)	39(0.3)	0.001
**VT/VF**	4(2.6)	16(3)	0.80	317(6.8)	267(2.3)	0.001
Advanced Heart Block*	4(2.6)	2(0.4)	0.009	150(3.2)	126(1.1)	0.001

Data are expressed in numbers (%) of patients. VT  =  ventricular tachycardia;

VF  =  ventricular fibrillation,* defined as 2^nd^ or 3^rd^ degree AV block.

#### Multiple logistics regression analysis

**Figure 2:**
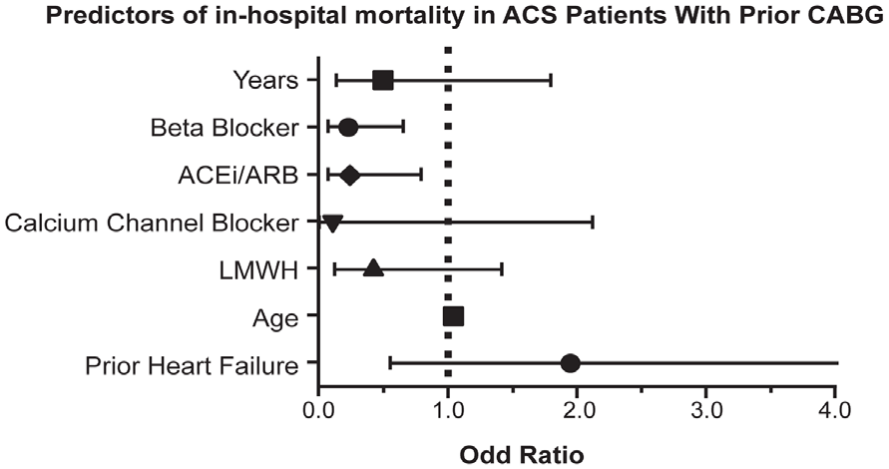
The predictors of in-hospital mortality in patients with prior coronary artery bypass surgery who presented with acute coronary syndrome. CABG  =  coronary artery bypass graft; ACS  =  acute coronary syndrome; ACE  =  angiotensin-converting enzyme; ARB  =  angiotensin receptor blocker; LMWH  =  low molecular weight heparin.

Multiple logistic regression revealed that prior heart failure (adjusted OR  = 1.94, 95% CI 1.18–3.24, P = 0.01) and age (adjusted OR = 1.05, 95% C.I. 1.03–1.06, P = 0.001) were risk factor for mortality. Prior CABG did not show statistical reduction in-hospital mortality (OR 0.55, 95% CI 0.21 to 1.41; p = 0.21) whereas, admission medication like, aspirin, B-blockers, CCBs, ACE inhibitors/ARBs and low molecular weight heparin (LMWH) were showed significant reduction in mortality rate (Table 6, Figure 2).

**Table 6:** Multivariate analysis of predictors of in-hospital mortality in patients presented with acute coronary syndromes.

Independent Predictor	Odds Ratio	95% C.I.	P value
**Patients characteristics**			
Male gender	0.69	0.43–1.08	0.11
Age	1.05	1.03–1.06	0.001
Current smoking	1.02	0.66–1.57	0.94
Diabetes mellitus	0.99	0.68–1.47	0.98
Hypertension	1.36	0.91–2.03	0.14
Chronic renal impairment	0.94	0.48–1.86	0.85
Prior myocardial infarction	1.06	0.67–1.67	0.81
Dyslipidemia	1.07	0.67–1.71	0.76
Prior heart failure	1.95	1.18–3.24	0.01
Body Mass Index	0.99	0.96–1.02	0.60
**In-hospital therapy**			
Thrombolytic therapy	0.77	0.46–1.27	0.30
Percutaneous coronary intervention	0.86	0.50–1.45	0.56
**In-hospital medication**			
Asprin	0.50	0.30–0.84	0.009
Clopidogrel	0.81	0.55–1.18	0.27
Beta blocker	0.23	0.15–0.35	0.001
Calcium channel blocker	0.11	0.03–0.34	0.001
ACEi/ARB	0.24	0.15–0.39	0.001
Unfractionated heparin	1.38	0.90–2.12	0.13
LMWH(enoxaparin)	0.42	0.26–0.69	0.001
**Prior CABG**	0.55	0.21–1.41	0.21

CABG  =  coronary artery bypass graft; LMWH  =  low molecular weight heparin; ACE =  angiotension converting enzyme, ARB  =  angiotensin receptor blocker; CI  =  confident interval.

## Discussion

The current study reports the clinical characteristics, treatment and outcome of Middle-eastern ACS patients according to their history of prior CABG. Patients with prior CABG were older, constitutes more females and carrying more cardiovascular risk factors including diabetes mellitus and hypertension than non-CABG group. Prior CABG patients were more likely to have larger infarct size and less likely to receive reperfusion therapy and early invasive therapy when compared to non-CABG patients. Despite having worse clinical profile, prior CABG patients had comparable morbidity and mortality to non-CABG patients. Over 20-years period, the current study reports for the *first* time significant reduction in mortality and stroke rates among ACS patients with prior CABG, which may be attributed to improvement in surgical CABG techniques and more use of evidence therapies.

The prevalence of prior CABG in ACS patients is variable. This study demonstrates a lower proportion of patients with prior CABG (4.2%) in our ACS cohort as compared to previous reports from Western countries (7%–27%), [8]–[10], [17]–[22]. However, similar data were reported in other series [5], [23], [24]. We reported an increasing number of CABG patients hospitalized with ACS matching the significant increase in the population of Qatar which nearly tripled since 2001 (600,000 population in 2001 and 1.6 million population in 2010).

### Clinical Characteristics

The reported older ages in CABG patients is consistent with previous studies when compared to patients without prior CABG [9], [18], [21], [25]–[27]. Likewise, the predominance of men in both groups. Females were significantly more prevalent in our population with prior CABG, a similar finding was reported in previous studies conducted on same Middle-eastern population [28], [29]. This female preponderance with prior CABG was not reported in Western population. In comparison to patients without prior CABG and consistent with the earlier reports, CABG patients clearly had more adverse baseline characteristics and burden of co-morbidities when presenting with ACS. They more often had a history of cardiovascular risk factors such as hypertension, diabetes and dyslipidemia, as well as, higher prevalence of chronic renal impairment. In addition, they had a greater likelihood of prior MI and treated heart failure [9], [18], [22], [23], [25]–[27], [30].

### In-hospital Management

Evidence-based therapies among ACS patients with prior CABG are very limited to post-hoc analysis of limited number of trials and registries, which suggested underuse of evidence-based therapies in this high-risk group as aspirin, beta-blocker, or thrombolytic therapy in the VALIANT trial [10], or evidence-based medications and PCI in the expanded-GRACE analysis in NSTEMI [3]. Our observations demonstrate increased use of aspirin, clopidogrel, β-blockers, ACE/ARB among ACS patients with prior CABG. The significant greater likelihood of evidence-based medications used before and during admission in our cohort with prior CABG reflect the unprejudiced utilization of evidence based medication in previous CABG patients by our physicians as well as this results may emphasize on the protective value accomplished by these medications as have been well established in different studies to reduce mortality in patients with coronary artery disease [31]–[35]. We observed an overall very low use of invasive procedures including coronary angiography and percutaneous coronary revascularization when compared to reports from Western countries, however it is consistent with recent study (2006–2007) conducted in 6 Middle –eastern countries and reported overall 20% use of coronary angiography among ACS patients [36].

### The Effect of Prior CABG on Outcomes

#### In-hospital mortality

The effect of prior CABG on mortality after ACS remains controversial (Table 7). Observational studies based on selected clinical trial populations have demonstrated a worse clinical outcomes and reported higher likelihood of adverse events of patients with ACS and prior CABG compared to those without prior CABG [10], [18], [19], [22], [37]–[39]. Prior CABG is an independent risk factor for in-hospital mortality [10] and at both 30 days and 1 year after MI [40], [41]. In GUSTO-1 (Global Utilization of Streptokinase and TPA for Occluded Arteries I) trial, patients with prior CABG had shown an increased 30-day mortality of 10.7% vs 6.7% (P = 0.001). In the GRACE registry prior CABG was associated with increased both in-hospital mortality and early mortality [7], [30]. All these older studies showed poor prognosis in patients with prior CABG presenting with ACS.

**Table 7:** Different studies compared prior coronary artery bypass surgery patients presenting with acute coronary syndrome.

Author	Name of Study	Year Published(Period ofStudy)	Total Number(% CABGpatients)	Type of ACS	Age of Prior CABG	Female withPrior CABG %	Mortality in Prior CABG Patients & Statistical Significance				
							In-hospitalmortality	30 daysmortality		3 month - ≥1 yearmortality	
Davis et al [17]	CASS Registry	1992 (5 years)	1,354 (27.2%)	MI	<45 (15%); 45–65(74%); >65 (11%)	21%	↓ (P<0.0001)	↓ (P<0.0001)		**–**	
Labinaz et al [25]	GUSTO-I	2001 (27 months)	41,021 (4%)	STEMI	Median 64.4(57, 70)	16.2%	**–**	↑ (P<0.001)		**–**	
Al Suwaidi et al [8]	The Mayo Clinic PTCA registry	2001 (7 years)	1,072 (11.9%)	MI	Mean 69.3±9.1	25%	**–**	**–**		↑ 1 year (P = 0.04)	
Peterson et al [20]	NRMI-2 Registry/United States	1999 (2 years)	45,925 (12.3%)	MI	Mean 64.6	20.2%	Prior CABG was independent predictor of mortality, (odds ratio 1.23; 95% confidence interval 1.05 to 1.44)				
Servoss et al [23]	PRISM-PLUS Trial	2004 (23 months)	1,570 (14.7%)	ACS	–	–	↓ At 48 hr: P = 0.09At 7 days: P = 0.035		↓ (P = 0.015)		↓ At 180 days: (P = 0.057)
Labinaz et al [24]	PURSUIT Trial	2002 (15 months)	9,455 (12%)	NSTE-ACS	Median 64(54, 71)	25%	**–**		↑ (P = 0.019)		↑ At 180 days (P = 0.021)
Kugelmass et al [22]	TACTICS-TIMI 18 Trial	2006 (2 years)	2,220 (22%)	NSTE-ACS	Mean 64.2	26%	**–**		**–**		↑ 6 months (P = 0.002)
Mathew et al [18]	NRMI-3 Registry	2002 (1 year)	112,697 (14.1%)	MI	Mean 71.4±10	STE/LBBB(29.6); NSTE/LBBB (30.3)	Unadjusted mortality; for STE/LBBB 16.2% vs 14.1%,; for NSTE/LBBB 10.1% vs 12.4% (P = 0.0001) weak association with adjusted in-hospital mortality in STE/LBBB (OR 1.11, 95% CI 1.00–1.23), but not in NSTE/LBBB (OR 0.99, 95% CI 0.92–1.07)				
Berry et al [19]	VALIANT Trial	2009 (2.5 years)	14,703 (7%)	MI	Mean 67±10	21%	**–**	**–**			↑ (*P = *0.0001)
Elbarasi et al [3]	ACS I, ACS II, and GRACE/expanded-GRACE Registries/Canada	2010 (8.5 years)	12,483 (9.6%CABG); (5% PCIand CABG	NSTE-ACS	CABG 72 y (63–78) PCI +/or CABG70yr (62–77)	23.5%	↓ (P<0.001)	**–**			**–**
Brilakis et al [21]	PROVE IT-TIMI 22 and A toZ Trials	2008 (14 months)	8,655 (7.4%)	ACS	Mean 64±10	17%	**–**	**–**			↑ 2 year (P<0.001)
Welsh et al [26]	APEX-AMI	2010 (2 years)	5,745 (2.2%)	STEMI withPrimary PCI	69 (58.3–76.0)	14.1%	**–**	**–**			↑ 90-day (P = 0.001)
Teixeira et al [5]	Prospective observational study	2010 (32 months)	1,495 (5.6%)	ACS	Mean 69.2 (9.4)	13.7%	No significant difference(P = 0.2)	No significantdifference(P = 0.87)			No significant difference(P = 0.87)
Alanbaei et al [25]	Gulf RACE Registry	2011 (1 year)	8,176 (5.6%)	ACS	Range 63 (56–70)	28.6%	↑ (P = 0.019)	**–**			**–**
Kim et al [4]	National Cardiovascular DataRegistry Acute Coronary Treatmentand Intervention Outcomes NetworkRegistry-Get With The Guidelines	2010 (1 year)	47,557 (18.5%)	NSTEMI	Median 72(63–80)	30%	No significantdifference	**–**			**–**
Nikolsky et al [44]	ACUITY Trial	–	13,774 (17.9%)	ACS	–	–	**–**	More death 1.8% vs1.5%,(P = 0.18)			**–**
Al-Aqeedi et al [24]	Gulf RACE-2 Registry	2011 (9 months)	7,881 (4.2%)	ACS	Mean 63.1±10.8	23.5%	No significant difference(P = 0.735)	No significant difference(P = 0.277)			No significant difference after1 year (P = 0.204)
Present Study	CCU Registry	(20 years)	16,750 (4.3%)	ACS	Mean 60.5±11	17.7%	No significant difference(P = 0.52)	**–**			*Trend of death over 20 years,* *reduced from 13.2% to 4%* *(p = 0.001)*

CABG  =  coronary artery bypass graft; MI  =  myocardial infarction; STEMI  =  ST elevation myocardial infarction; NSTEMI  =  non ST-elevation myocardial infarction; LBBB  =  left bundle branch block; ACS  =  acute coronary syndrome; C.I. =  confidence interval; H.R. = hazard ratio.

On the other hand, more recently several studies have showed no significant differences in in-hospital and long term prognosis in patients with or without prior CABG presenting with ACS. Teixeira et al, reported no significant influence of prior CABG on short or medium term outcomes, such as all-cause mortality and adverse cardiac events in patient presenting with ACS [5]. As well as, Kim et al [4] concluded that, in-hospital mortality did not differ significantly from non-CABG patients who had NSTEMI (adjusted ORs 1.00, 95% CI 0.92–1.11 and 0.99, 95% CI 0.87–1.11, respectively). In addition, Elbarasi et al, demonstrated less in-hospital mortality in NSTE-ACS patient with prior CABG (1.7%) and prior CABG with PCI (0.9%) than in patients without prior CABG (2.3%) (P = 0.001) [3]. Wisemann et al, reported similar 1- and 5-year mortality rates in patients with and without prior CABG [42]. Furthermore, Mathew et al, reported a weak association of prior CABG with adjusted in-hospital mortality in patients who had STE/LBBB (OR 1.11, 95% CI 1.00–1.23), but not in patients had NSTE/LBBB (OR 0.99, 95% CI 0.92–1.07), [9]. These reports may point toward a possible protective role might be played by CABG against death in patients who have history of severe coronary artery disease enough to merit revascularization presented currently with ACS. In addition, the better prognosis observed in the recent studies may be a sign toward the role of the guideline recommended evidence based therapies in the management of coronary artery disease patients.

In this study it was found that although patients who had previously undergone CABG presented with significantly adverse baseline characteristics and had larger infarct size, they demonstrate no significant difference in in-hospital mortality when compared to patients without prior CABG. It remains somewhat intriguing, why high risk background did not translate into worse mortality. Advanced age is an important risk factor for mortality, however our CABG patients demonstrated to be younger than other reported cohorts. This along with less recurrence of ischemia or infarction together with the higher prevalence of previous and in-hospital treatment with evidence-based medications may explain to some extent their favorable outcome.

### The Effect on Stroke and other Morbidities

Most of studies revealed a higher prevalence of prior stroke in prior CABG patient when presented with ACS. However, the occurrence of stroke as a new complication in those patients is still controversial.The observed non-significant difference of stroke between post-CABG patients and those who had not undergone a previous CABG, was a similar finding observed by texiera et al [5] and others [18]. In contrary brikalis et al reported those with previous CABG were approximately 2 times as likely as to develop stroke than those without prior CABG [19]. Likewise, Berry et al, showed CABG patients were more likely to experience the composite outcome of cardiovascular death, MI, HF, resuscitated cardiac arrest, or stroke; 3 year Kaplan–Meier rate, 64 vs. 39% (adjusted hazard ratio 1.29, 95% CI 1.17–1.43; P, 0.0001), [10].

### The Trends

Mortality rates from coronary heart disease and from acute MI, in particular, have been declining steadily since the 1970s in several Western populations. In the United States, Krumholz et al, reported significant decrease in the risk-standardized hospital mortality rate for patients discharged with Acute MI between 1995 and 2006 [43]. We observed for the first time, over 20 year period, there was a significant reduction in mortality and stroke rates among our population with or without prior CABG who presented with ACS.

It is worth mentioning that, although, the results were generalized and including all patients admitted to CCU and no case exempted for registration in the registry. The result of this study should be interpreted in the context that, the data presented were intended to provide a fairly general representation of the practice and trends over the last 2 decades; as such, these data are largely descriptive without adjustment for case mix, so conclusions based on observed associations should be made with caution.

### Conclusion

The current study on the clinical characteristics, treatment and outcome of Middle-eastern ACS patients according to the history of prior CABG reports significant reduction in-hospital morbidity and mortality among ACS patients with or without prior CABG over 20-years period.

### Study Limitations

First, as in all registry studies spanning long time intervals, admission policies to the CCU and management of ACS would have changed considerably during this study period. Second, our data were collected retrospectively from records registration over 20 years, which is another limitation. The fundamental limitations of retrospective observational studies cannot be eliminated because of the nonrandomized nature and unmeasured confounding factors. However, well-designed retrospective observational studies may provide valid results without systematic overestimation, bias or predilection. Finally, detailed surgical data was not available on prior CABG patients such as the number of bypass grafts and the use arterial grafts.
